# P-14. Unmasking the Enemy: Clinical and Microbiological Insights into P. aeruginosa Bacteremia

**DOI:** 10.1093/ofid/ofaf695.245

**Published:** 2026-01-11

**Authors:** David S Burgess, Katie B Olney, Donna R Burgess

**Affiliations:** University of Kentucky, Lexington, KY; University of Kentucky HealthCare, Lexington, KY; UK HealthCare, Lexington, KY

## Abstract

**Background:**

*P. aeruginosa* bacteremia presents significant treatment challenges due to resistance patterns and high mortality. This study aimed to characterize the clinical features, antimicrobial therapy, and outcomes associated with *P. aeruginosa* bacteremia and identify predictors of in-hospital mortality

**Methods:**

This retrospective cohort study included all inpatients with an initial episode of *P. aeruginosa* bacteremia at UK HealthCare between July 2022 and December 2024. Of 970 patients with positive blood cultures for monomicrobial Gram-negative bacteria, 91 (9.4%) had *P. aeruginosa* bacteremia. Data were obtained through structured chart review and the Center for Clinical and Translational Science. Univariate analysis using SPSS identified mortality predictors.

**Results:**

Median (IQR) age was 66 yrs (50, 74); 87.9% were White. Comorbidities included chronic kidney disease (46.2%), peripheral vascular disease (44.0%), COPD (37.4%), and diabetes (24.2%). Infections were hospital-acquired in 51.6%, and 56.0% required ICU admission. In-hospital mortality was 30.8%, and 47.3% were readmitted within 90 days. Median hospital and ICU stays were 17 (8, 32) and 7.0 days (4.1, 14.3), respectively.

Initial empiric therapy included cefepime (CFP, 48.3%), piperacillin/tazobactam (PTZ, 27.5%), meropenem (MER, 9.9%), ceftolozane/tazobactam (C/T, 2.2%), and miscellaneous agents (12.1%). Definitive therapy reflected similar trends: CFP (44.0%), PTZ, MER, and miscellaneous (16.5% each), and C/T (6.5%). Carbapenem resistance was 15%. ID consultation occurred in 57.1% of cases, with no difference between ICU and non-ICU patients (54.9% vs 60.0%, p=NS). Mortality was associated with ICU admission (p=0.004), qPITT (p=0.006), SOFA (p=0.011), hospital-acquired infection (p=0.039), and indwelling catheter (p=0.046).

**Conclusion:**

Among patients with Gram-negative bacteremia, *P. aeruginosa* accounted for 9.4% of cases and was associated with high mortality, frequent ICU admission, and prolonged hospitalization. CFP and PTZ were the most common therapies; novel agent use was limited. Mortality was linked to critical illness and nosocomial acquisition. Findings support timely risk assessment, ID consultation, and optimized therapy.

**Disclosures:**

All Authors: No reported disclosures

